# Genetic Interaction Effects of Heading Date Genes *Hd1* and *Ghd7* on Photosynthetic Traits at the Heading Stage in Rice

**DOI:** 10.3390/plants15060977

**Published:** 2026-03-22

**Authors:** Jun Shi, Yi-Jie Yan, Zhen-Hua Zhang, Ye-Yang Fan, De-Run Huang, Yu-Jun Zhu, Bo Shen

**Affiliations:** 1 College of Life and Environmental Sciences, Hangzhou Normal University, Hangzhou 311121, China; sj247919@163.com (J.S.); 2023111010029@stu.hznu.edu.cn (Y.-J.Y.); 2 State Key Laboratory of Rice Biology and Breeding, China National Rice Research Institute, Hangzhou 310006, China; zhangzhenhua@caas.cn (Z.-H.Z.); fanyeyang@caas.cn (Y.-Y.F.);

**Keywords:** photosynthesis, heading date, chlorophyll content, *Heading date 1*, *Grain number, plant height, and heading date 7*, *Oryza sativa* L.

## Abstract

In this study, we dissect the genetic effects of two major rice heading date genes, *Heading date 1* (*Hd1*) and *Grain number, plant height, and heading date 7* (*Ghd7*), in the regulation of six photosynthesis-related traits: the chlorophyll *a*/*b* contents, net photosynthetic rate (Pn), stomatal conductance (Gs), intercellular CO_2_ concentration (Ci), and transpiration rate (Tr). Using two sets of near-isogenic lines (Z43 and Z44) derived from a Zhenshan97/Milyang46 cross, functional *Hd1* increased the chlorophyll contents but decreased the photosynthesis-related parameters; however, functional *Ghd7* consistently inhibited all six traits. More importantly, there is a significant epistatic interaction between them: *Hd1* only enhances the photosynthetic capacity under the non-functional background of *ghd7* but intensifies its photosynthesis inhibition under the functional background of *Ghd7*. Transcriptome analysis showed that functional *Ghd7* mainly down-regulated the expression of genes related to photosynthesis and chloroplast development, and the inhibitory effect was significantly enhanced in the presence of functional *Hd1*. GO enrichment analysis further confirmed that the chlorophyll synthesis, photosystem assembly, and electron transfer pathways were downregulated in the bifunctional allele combination. Although *Hd1* promotes chlorophyll accumulation, it reduces the actual photosynthetic efficiency, indicating that it has different regulatory paths for chlorophyll synthesis and photosynthetic function. Both physiological and molecular evidence showed that the *Hd1*-*Ghd7* module coordinated the regulation of the heading date and photosynthetic capacity, forming a trade-off relationship between “early heading–high photosynthesis” and “late heading–low photosynthesis”. This study reveals the pleiotropy of genes at the heading stage and provides a theoretical basis for the optimization of the source–sink balance in high-yield rice breeding.

## 1. Introduction

Both the heading date (HD) and photosynthesis are potential factors affecting rice yields [1,2,3], and there is a certain relationship between them in controlling rice growth, development, flowering, and reproduction [4]. Mining and utilizing the genes controlling the HD and photosynthesis can effectively improve rice yields [5,6,7]. After nearly a decade of scientific research, scientists have clearly revealed two basic pathways regulating the HD: (1) the *OsGI*–*Hd1*–*Hd3a* pathway with *Hd1* as the core gene, and (2) the *Ghd7*–*Ehd1*–*RFT1*/*Hd3a* pathway with *Ehd1* as the core gene [8,9,10,11]. These two pathways basically control the diversity of the HD. The bifunctional action of *Hd1* has been well recognized, promoting flowering under the short-day (SD) condition and inhibiting flowering under the long-day (LD) condition [12], and *Ghd7* is an important negative regulator of the pathway with *Ehd1* as the core on the HD under the LD condition [13]. *Hd1* and *Ghd7* are pleiotropic genes that control heading dates and rice grain yields.

Studies have also shown that *Hd1* and *Ghd7* interact with each other: *Hd1*-dependent regulation under the LD condition depends on *Ghd7*, which delays heading under the *Ghd7* background and promotes heading under the *ghd7* background. Moreover, the delay effect of *Ghd7* on heading is significantly amplified under the *Hd1* background. This heading stage regulation process is achieved through the formation of a functional CCT/NF-YB/YC protein complex via the interaction of *Hd1* and *Ghd7* with other heading stage regulating factors, such as *DTH8* and *Hd2* [14].

The chlorophyll contents, net photosynthetic rate (Pn), stomatal conductance (Gs), intercellular CO_2_ concentration (Ci), and transpiration rate (Tr) are key physiological indicators for assessing the photosynthetic capacity of plants. The chlorophyll contents are a physiological trait that is directly related to photosynthesis efficiency [15], and previous studies have demonstrated that HD genes exhibit significant genetic effects on them. Functional *Ghd7* reduces chlorophyll accumulation by repressing the expression of genes involved in chlorophyll and chloroplast biosynthesis [16]. Similarly, *Ghd8*/*DTH8*/*LHD1* is a pleiotropic regulator of both the heading date and chlorophyll contents, and it localizes to the genomic region harboring the major chlorophyll content QTL *qFCC8L*. Like *Ghd7*, *Ghd8* negatively regulates chlorophyll biosynthesis genes to decrease chlorophyll levels [17,18]. Another major chlorophyll content QTL, *qSCC6IL*/*qTCC6IL*, co-localizes with the photoperiod-sensitive heading date gene *Se5*/*OsHY1*/*OsHO1*, which encodes a heme oxygenase essential for phytochrome chromophore biosynthesis. Loss-of-function *se5* mutants exhibit elevated *Ehd1* and *Hd3a* expression, resulting in early flowering and concomitant chlorophyll content alterations [19]. Moreover, *se5* affects chlorophyll synthesis and is essential for early chloroplast synthesis [20,21]. *Se13*/*OsHY2* encodes a phytochromobilin synthase and displays photoperiod sensitivity, it genetically interacts with both *Hd1* and *Ghd7* to modulate the heading date, and its functional loss abolishes *Hd1*-mediated photoperiod response [22]. The non-functional *se13* allele significantly reduces chlorophyll *a*/*b* contents, leading to a diminished photosynthetic capacity, a lower 1000-grain weight, and an impaired grain-filling rate [23]. *Ghd7.1*/*DTH7*/*OsPRR37* is a pleiotropic gene governing plant height, heading date, grain yield, chlorophyll content, and photosynthetic capacity [24,25,26].

The aforementioned studies demonstrate that the heading date gene exerts pleiotropic effects on the chlorophyll contents. Consistent with this finding, several studies have reported that chlorophyll synthetase participates in both light signal transduction and circadian rhythm regulation. *YGL1*, encoding chlorophyll synthase, regulates the expression of *OsCAB1R* and *OsCAO*, and *OsCAB1R* encodes a light-harvesting chlorophyll *a*/*b*-binding protein whose transcription is under circadian control; reciprocally, optimal circadian clock function requires proper *OsCAB1R* expression [27]. *OsCAO* encodes chlorophyll *a* oxygenase, with two paralogs, *OsCAO1* and *OsCAO2*, both subject to light and circadian regulation [28]. *OsCAO1* over-expression increased chlorophyll *b* synthesis, and the over-expression plants had a higher photosynthetic rate and heavier 1000-grain weight than the wild-type variety Zhefu 802 [29].

Relative to the research progress regarding the HD gene regulation of chlorophyll contents, genetic effects of HD genes on the other four photosynthetic traits remain poorly documented. Moreover, prior studies predominantly focused on the additive effects of individual heading date (HD) genes on chlorophyll accumulation, whereas epistatic interactions between HD genes—and their functional impact on chlorophyll contents—remain poorly characterized. In this study, two near-isogenic line sets derived from an *indica* rice cross between the maintainer line Zhenshan97 (ZS97) and the restorer line Milyang46 (MY46) were employed to dissect the genetic effects of *Hd1*, *Ghd7*, and their interactions on the six photosynthesis-related traits. Concurrently, transcriptome profiling and GO enrichment analysis were conducted on NILs harboring distinct *Hd1*-*Ghd7* allelic combinations to characterize their impact on photosynthesis-related regulatory pathways. These results provide a reference for understanding how HD genes regulate photosynthesis.

## 2. Results

### 2.1. Effects of Hd1 and Ghd7 on Photosynthesis-Related Traits in Z44 Population

**Figure 1:**
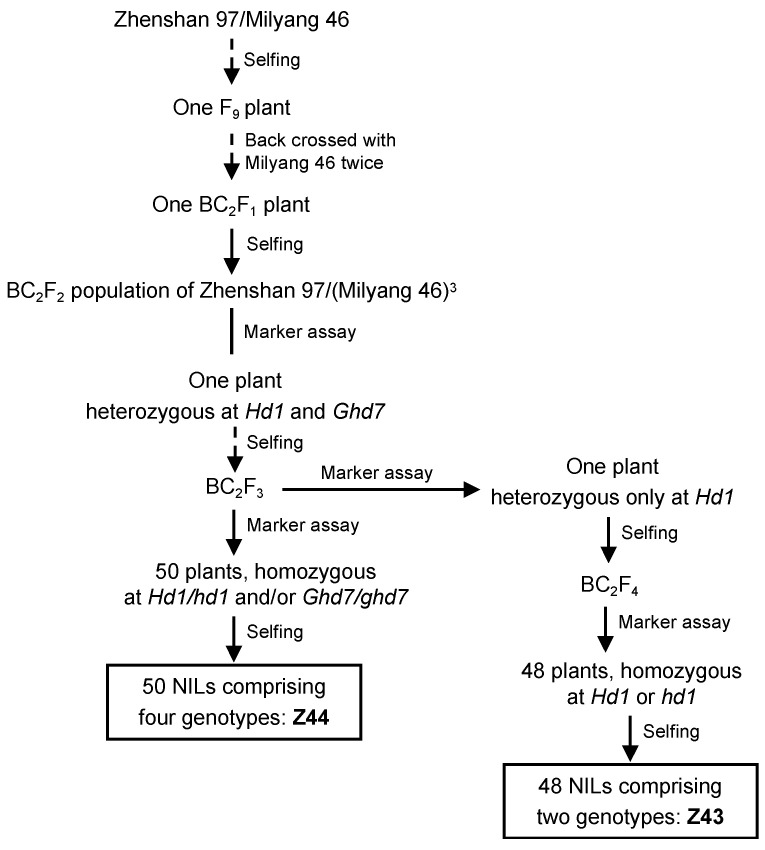
The development of the rice populations used in this study. NIL, near-isogenic line.

The effects of *Hd1* and *Ghd7* on the photosynthesis-related traits were investigated using the near-isogenic line (NIL) population Z44, which was developed from the rice cross ZS97/MY46//MY46///MY46 and segregates for both *Hd1* and *Ghd7* loci (Figure 1). The female parent, ZS97, carries the functional *Hd1* allele and the non-functional *ghd7* allele, whereas the male parent, MY46, carries the non-functional *hd1* allele and the functional *Ghd7* allele. The Z44 population comprised four homozygous genotypic combinations of *Hd1* and *Ghd7*, i.e., *hd1*^MY46^*ghd7*^ZS97^ (NN), *Hd1*^ZS97^*ghd7*^ZS97^ (FN), *hd1*^MY46^*Ghd7*^MY46^ (NF), and *Hd1*^ZS97^*Ghd7*^MY46^ (FF), respectively. “F” = functional allele; “N” = non-functional allele. Under LD conditions, six physiological traits were measured at heading: the net photosynthetic rate (Pn), stomatal conductance (Gs), intercellular CO_2_ concentration (Ci), transpiration rate (Tr), chlorophyll *a* content, and chlorophyll *b* content. These parameters collectively reflect plant photosynthetic capacity and efficiency.

The effects of these two genes on the six traits are shown in Table 1. For the chlorophyll contents, the genetic effects of *Hd1* and *Ghd7* were significant, but their interaction was not. The functional *Hd1* allele from ZS97 increased the chlorophyll *a*/*b* contents by 0.07 and 0.04 mg/g·FW, respectively, accounting for 9.18% and 13.98% of the phenotypic variation, respectively, while the functional *Ghd7* allele from MY46 decreased the chlorophyll *a*/*b* contents by 0.13 and 0.02 mg/g·FW, respectively, accounting for 14.32% and 6.22% of the phenotypic variation, respectively.

For the remaining four traits, the effects of *Ghd7* and the digenic interaction were significant, but the effects of *Hd1* were not. The additive effects of *Ghd7* on the Pn, Gs, Ci, and Tr were estimated to be 3.31 μmol CO_2_ m^−2^ s^−1^, 0.19 mol H_2_O m^−2^ s^−1^, 42.99 μmol CO_2_ m^−2^ s^−1^, and 1.78 mmol H_2_O m^−2^ s^−1^, respectively, and the *R*^2^ values were estimated to be 35.14%, 43.15%, 53.13%, and 40.37%, respectively. The functional *Ghd7* allele from MY46 decreased the four photosynthetic traits.

**Figure 2:**
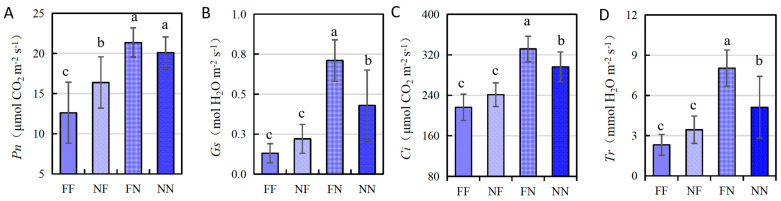
Interaction effects of *Hd1* and *Ghd7* on photosynthetic traits. (**A**–**D**) Interaction effects of *Hd1* and *Ghd7* on Pn, Gs, Ci, and Tr, respectively. FF: *Hd1*^ZS97^*Ghd7*^MY46^ genotype; NF: *hd1*^MY46^*Ghd7*^MY46^ genotype; FN: *Hd1*^ZS97^*ghd7*^ZS97^ genotype; NN: *hd1*^MY46^*ghd7*^ZS97^ genotype; data are represented as mean ± SD; bars with different letters are significantly different at *p* < 0.01 based on Duncan’s multiple range tests.

The *R*^2^ values of the *Hd1* and *Ghd7* interaction were estimated to be 5.58%, 7.84%, 6.69%, and 10.70% for the Pn, Gs, Ci, and Tr, respectively (Table 2). Compared with the NILs with the NN genotype, increases were observed in the FN genotype for all four traits, although they did not reach a statistically significant level for the Pn (Figure 2). The Pn, Gs, Ci, and Tr increased by 1.25 μmol CO_2_ m^−2^ s^−1^, 0.28 mol H_2_O m^−2^ s^−1^, 35.43 μmol CO_2_ m^−2^ s^−1^, and 2.92 mmol H_2_O m^−2^ s^−1^, respectively. Compared with the NILs with the NF genotype, decreases were observed in the FF genotype for all four traits, although they did not reach significant levels for the Gs and Tr. The Pn, Gs, Ci, and Tr decreased by 3.77 μmol CO_2_ m^−2^ s^−1^, 0.09 mol H_2_O m^−2^ s^−1^, 25.03 μmol CO_2_ m^−2^ s^−1^, and 1.13 mmol H_2_O m^−2^ s^−1^, respectively. These results indicate that the functional *Hd1* allele increased the photosynthetic capacity during the heading date in the non-functional *ghd7* background but decreased the photosynthetic capacity in the functional *Ghd7* background.

Compared with the NILs with the FN genotype, the Pn, Gs, Ci, and Tr values of the FF genotype decreased by 8.74 μmol CO_2_ m^−2^ s^−1^, 0.58 mol H_2_O m^−2^ s^−1^, 115.43 μmol CO_2_ m^−2^ s^−1^, and 5.72 mmol H_2_O m^−2^ s^−1^, respectively, and compared with the NILs with the NN genotype, the Pn, Gs, Ci, and Tr of the NF genotype decreased by 3.72 μmol CO_2_ m^−2^ s^−1^, 0.21 mol H_2_O m^−2^ s^−1^, 54.97 μmol CO_2_ m^−2^ s^−1^, and 1.67 mmol H_2_O m^−2^ s^−1^, respectively. According to these results, the functional *Ghd7* allele reduced the four photosynthetic traits regardless of the *Hd1* genotype, but its effect was enhanced by the functional *Hd1* allele.

### 2.2. Effects of Hd1 on Photosynthesis-Related Traits in Z43 Population

**Figure 3:**
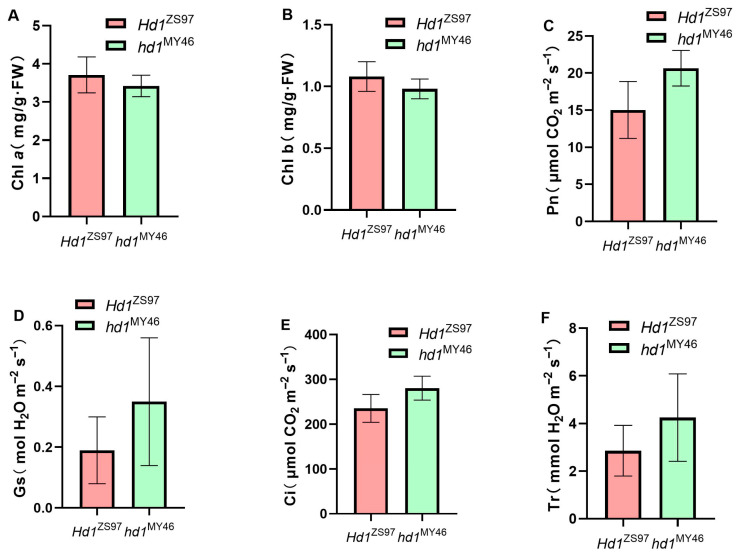
The phenotypic differences for the six photosynthesis-related traits at the *Hd1* locus in the Z43 population. (**A**) Chl *a*, chlorophyll *a* (mg/g·FW); (**B**) Chl *b*, chlorophyll *b* (mg/g·FW); (**C**) Pn, photosynthesis rate (μmol CO_2_ m^−2^ s^−1^); (**D**) Gs, stomatal conductance (mol H_2_O m^−2^ s^−1^); (**E**) Ci, intercellular CO_2_ concentration (μmol CO_2_ m^−2^ s^−1^); (**F**) Tr, transpiration rate (mmol H_2_O m^−2^ s^−1^); data are represented as mean ± SD; *n* = 22 for *Hd1*^ZS97^; *n* = 26 for *hd1*^MY46^.

To further analyze the genetic effects of *Hd1* on these six traits, another population, Z43, was constructed (Figure 1). This population segregates only at the *Hd1* locus, which comprises 26 *hd1*^MY46^ lines and 22 *Hd1*^ZS97^ lines. Phenotypic differences for the six photosynthesis-related traits at the *Hd1* locus in the Z43 population are shown in Figure 3. As expected, the chlorophyll contents of the *Hd1*^ZS97^ lines are higher than those of the *hd1*^MY46^ lines (Figure 3A,B); however, the phenotypic values of the other four traits are lower than those of the *hd1*^MY46^ lines (Figure 3C–F). The results of the analysis of variance are shown in Table 2. All six traits show significant differences between the lines carrying the functional *Hd1* and non-functional *hd1* alleles. The functional *Hd1* allele from ZS97 decreased the four photosynthetic parameters, the Pn, Gs, Ci, and Tr, by 2.81 μmol CO_2_ m^−2^ s^−1^, 0.08 mol H_2_O m^−2^ s^−1^, 22.49 μmol CO_2_ m^−2^ s^−1^, and 0.69 mmol H_2_O m^−2^ s^−1^, respectively, and the *R*^2^ values were 31.90%, 10.77%, 21.19%, and 11.22%, respectively. The functional *Hd1* allele from ZS97 increased the chlorophyll *a*/*b* contents by 0.15 mg/g·FW and 0.05 mg/g·FW, respectively, and the *R*^2^ values were 17.60% and 27.62%, respectively. These results indicate that *Hd1* had significant genetic effects on these six photosynthesis-related traits under the homozygous *Ghd7* background.

### 2.3. RNA-Seq-Based Analysis of Hd1 and Ghd7 Expression Regulation in NILs

NILs harboring distinct *Hd1*-*Ghd7* genotype combinations from the Z43 and Z44 populations were subjected to RNA-seq analysis to further investigate the transcriptional regulation of *Hd1* and *Ghd7*. The differentially expressed genes (DEGs) identified in each pairwise comparison are summarized in Table 3. The *OsGI*, *Hd1*, *Ghd7*, *Ehd1*, *RFT1*, and *Hd3a* genes—involved in the two heading date regulation pathways—exhibited no significant expression differences in any comparison group, except for *Hd3a* in the Z43 population.

Table 3 shows that there were 1805 DEGs caused by functional *Hd1* in the Z43 population, of which 523 were up-regulated and 1282 were down-regulated. In the Z44 population, four *Hd1*-*Ghd7* genotype combinations were represented: *Hd1*^ZS97^*Ghd7*^MY46^ (FF), *Hd1*^ZS97^*ghd7*^ZS97^ (FN), *hd1*^MY46^*Ghd7*^MY46^ (NF), and *hd1*^MY46^*ghd7*^ZS97^ (NN). A total of 2836 DEGs were identified in the FF vs. FN comparison; relative to the FN line, 1916 genes were downregulated and 920 were upregulated in the FF line. In the NF vs. NN comparison, 1097 DEGs were detected; relative to the NN line, 621 genes were downregulated and 476 were upregulated in the NF line, which carries the functional *Ghd7* allele. Notably, the number of DEGs in the FF vs. FN comparison (conducted in a functional *Hd1* background) was approximately 3.0-fold higher than that in the NF vs. NN comparison (conducted in a non-functional *Hd1* background). Moreover, under functional *Hd1* conditions, the number of genes downregulated by functional *Ghd7* substantially exceeded the number of those upregulated, suggesting that *Ghd7*-mediated transcriptional repression is the predominant regulatory mode of functional *Ghd7*. Collectively, these results indicate that the regulatory impact of functional *Ghd7* is contingent upon the allelic status of *Hd1*.

A total of 564 DEGs were identified in the FN vs. NN comparison; relative to the NN line, 262 genes were downregulated and 302 were upregulated in the FN line. In contrast, the FF vs. NF comparison yielded 2082 DEGs; relative to the NF line, 1345 genes were downregulated and 737 were upregulated in the FF line. Notably, the number of DEGs in the FF vs. NF comparison (conducted in a functional *Ghd7* background) was approximately 3.7-fold higher than that in the FN vs. NN comparison (conducted in a non-functional *ghd7* background). Furthermore, under functional *Ghd7* conditions, functional *Hd1* induced nearly twice as many downregulated genes as up-regulated ones. By contrast, in the absence of functional *Ghd7* (i.e., in the FN vs. NN comparison), the numbers of *Hd1*-dependent down- and upregulated genes were comparable. Collectively, these findings demonstrate that the transcriptional regulatory activity of *Hd1* is dependent on the presence of a functional *Ghd7* allele.

### 2.4. Gene Ontology (GO) Functional Enrichment Analysis of Differentially Expressed Genes

**Figure 4:**
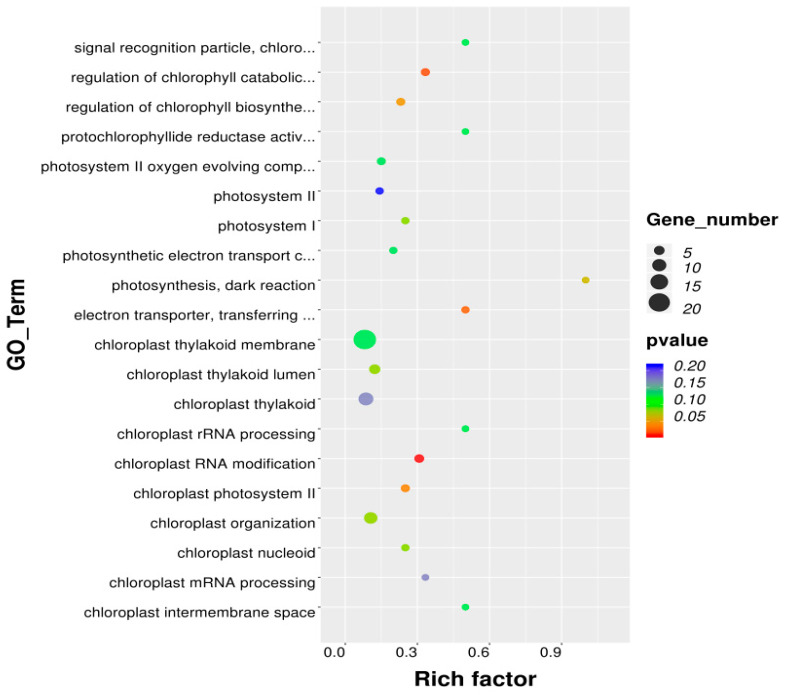
The Gene Ontology functional enrichment of the DEGs from the *Hd1*^ZS97^ vs. *hd1*^MY46^ group in Z43. Only the top 20 statistically significant GO terms associated with photosynthesis and chloroplasts are shown.

Five GO terms significantly enriched in biological processes associated with photosynthesis and chloroplast development were identified in the FF vs. NF comparison within the Z43 population (Figure 4): GO:001900865 (chloroplast RNA modification), GO:0010271 (regulation of the chlorophyll catabolic process), GO:00045156 (electron transporter activity), GO:0030095 (chloroplast photosystem II), and GO:0010380 (regulation of the chlorophyll biosynthetic process). These five GO terms collectively encompassed eight DEGs (Table S1), and clustering revealed that seven of these eight DEGs were downregulated in the FF line relative to the NF line, whereas *LOC_Os07g02280* was upregulated. These findings indicate that functional *Hd1* predominantly exerts transcriptional repression on genes involved in chloroplast function and photosynthetic machinery—consistent with the negative regulatory effect inferred from physiological trait analyses.

**Figure 5:**
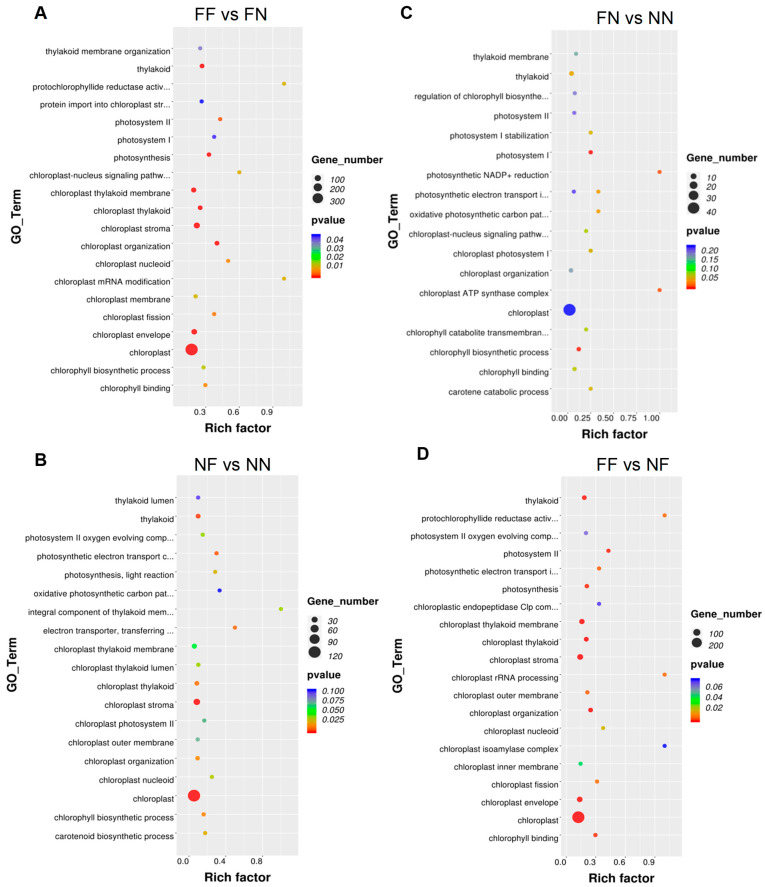
The Gene Ontology functional enrichment of the DEGs from the four *Hd1*-*Ghd7* genotype combinations in Z44: (**A**) FF vs. FN; (**B**) NF vs. NN; (**C**) FN vs. NN; (**D**) FF vs. NF. Only the top 20 statistically significant GO terms are shown. FF, *Hd1*^ZS97^*Ghd7*^MY46^; FN, *Hd1*^ZS97^*ghd7*^ZS97^; NF, *hd1*^MY46^*Ghd7*^MY46^; NN, *hd1*^MY46^*ghd7*^ZS97^.

GO functional enrichment analysis was conducted for the FF vs. FN and NF vs. NN comparisons within the Z44 population. The top enriched GO terms—such as nucleus, chloroplast, cytoplasm, protein binding, and ATP binding—largely overlapped between the two comparisons; however, the number of DEGs assigned to each shared term varied substantially. Notably, GO terms significantly associated with photosynthesis and chloroplast development totaled 20 in the FF vs. FN comparison and 14 in the NF vs. NN comparison (Figure 5A,B). Both comparisons annotated DEGs implicated in photosynthetic function and chloroplast biology, including *LOC_Os05g49920* (*OsPPR6*), *LOC_Os03g45400* (*OsNUS1*), *LOC_Os03g27770* (*OsHO2*), and *LOC_Os01g01340* (*OsLIR1*)—all of which exhibited down-regulated expression. In contrast, the FF vs. FN comparison uniquely annotated several key photosynthetic genes absent from the NF vs. NN results, including *LOC_Os02g02860* (*OsGluRS*), *LOC_Os04g58200* (*OsPORA*), and *LOC_Os10g35370* (*OsPORB*) (Table S2). Clustering analysis revealed that approximately two-thirds of the DEGs associated with photosynthesis were downregulated in both the FF vs. FN and NF vs. NN comparisons. These findings indicate that functional *Ghd7* exerts a predominant negative regulatory effect on photosynthetic processes and chlorophyll metabolism. Moreover, the regulatory impact of functional *Hd1* on these pathways is mediated through its genetic interaction with *Ghd7*—a conclusion corroborated by physiological trait analyses.

GO functional enrichment analysis was also conducted for the FN vs. NN and FF vs. NF comparisons. Strikingly, the sets of photosynthesis-related GO terms significantly enriched in these two comparisons were markedly distinct: only four terms were enriched in FN vs. NN, whereas seventeen terms—largely overlapping with those identified in the FF vs. FN comparison—were enriched in FF vs. NF (Figure 5C,D). Similarly, the key photosynthetic genes annotated under these enriched GO terms in the FF vs. NF comparison closely resembled those in the FF vs. FN comparison (Table S2). Clustering revealed no coherent expression pattern among DEGs in the FN vs. NN comparison; in contrast, DEGs in the FF vs. NF comparison exhibited a strong coordinated down-regulation—mirroring the trend observed in FF vs. FN. Collectively, these results demonstrate that the functional *Hd1*-mediated repression of photosynthetic gene expression is strictly dependent on the presence of a functional *Ghd7* allele—a conclusion fully supported by physiological trait analyses.

## 3. Discussion

In our previous study, we characterized the effects of the genetic interactions between *Hd1* and *Ghd7* on the heading date and yield-related traits in the Z44 population [30]. Comparative analysis with photosynthesis-related traits revealed that *Hd1* and *Ghd7* coordinately modulate the photosynthetic rate in a heading date-dependent manner: they suppress photosynthesis when delaying heading and enhance it when promoting heading (Figure 2). Among the four genotypic combinations, FN (75.0 d) exhibited the earliest heading date, followed by NN (78.5 d), NF (83.7 d), and FF (92.5 d). Correspondingly, their photosynthetic rates declined progressively: 21.4, 20.1, 16.4, and 12.6 μmol CO_2_ m^−2^ s^−1^, respectively. This inverse correlation between the heading date and photosynthetic capacity suggests that *Hd1*–*Ghd7* epistatic interaction imposes a trade-off between developmental timing and photosynthetic efficiency.

The relationship between the HD and grain yield per plant (GY) was analyzed using the four genotype combinations in the Z44 population in our previous study [30]. Under the SD condition, the HD range from earliest to latest was as follows: FN: 87.2 d, NN: 91.2 d, NF: 103.0 d, and FF: 108.6 d. The GY trend was consistent with that of the HD, increasing from lowest to highest as follows: FN: 20.7 g, NN: 24.0 g, NF: 29.4 g, and FF: 29.9 g. Under LD conditions, the HD still ranged from earliest to latest, as follows: FN: 75.0 d, NN: 78.5 d, NF: 83.7 d, and FF: 92.5 d. However, the GY trend followed a different pattern, as follows: FN: 30.4 g, NN: 30.5 g, NF: 30.9 g, and FF: 29.9 g. These results suggest that the grain yield may increase with delayed heading under the SD condition, but it does not necessarily increase with delayed heading under the LD condition. Notably, under the LD condition, the highest grain yield was observed not in the latest-heading FF line (GY: 29.9 g) but in the NN line (GY: 30.5 g)—despite its intermediate heading date—indicating that maximal yield does not necessarily coincide with maximal vegetative duration. These findings highlight the critical importance of temporally coordinating photosynthetic activity with reproductive development to maximize carbon assimilation and thereby enhance rice grain yields. Moreover, the results demonstrate that extending the growth duration—intended to capitalize more fully on available thermal and light resources—does not invariably increase grain yields. The regulatory mechanisms governing *Hd1* and *Ghd7* expression—and their functional impact on the source–sink balance in rice—remain to be fully elucidated.

The dual function of *Hd1* has been well recognized, promoting heading under SD conditions and inhibiting it under LD conditions [12]. In the Z43 population, the heading date of the *Hd1*^ZS97^ lines was later than that of the *hd1*^MY46^ lines [30], indicating that the functional *Hd1* allele delays heading, which is consistent with previous research results [31]. In the measurement of the photosynthesis-related traits, the four photosynthetic values of the *Hd1*^ZS97^ lines in the Z43 population were all lower than those of the *hd1*^MY46^ lines (Table 2), suggesting that functional *Hd1* reduces the photosynthetic capacity at the heading stage. Although the chlorophyll content is widely regarded as a key determinant of the photosynthetic performance, its relationship with the photosynthetic rate is not strictly linear. While numerous studies report a positive correlation between the total chlorophyll content and photosynthetic rate [32], emerging evidence suggests context-dependent complexity: Zhu et al. demonstrated that excessive chlorophyll accumulation can induce photosystem saturation, exacerbating photodamage and ultimately reducing the photosynthetic rate [33]; similarly, Ort et al. proposed that optimizing the antenna complex content—not maximizing the chlorophyll content—enhances the light-use efficiency and elevates the photosynthetic rate [34]. In the Z43 population, the functional *Hd1* allele increased the chlorophyll *a*/*b* contents but decreased the Pn, Gs, Ci, and Tr, indicating that *Hd1* has different genetic regulatory effects on photosynthesis and the chlorophyll contents.

Our previous study showed that the functional *Hd1* allele promotes heading when *ghd7* is non-functional but inhibits heading when *Ghd7* is functional. Functional *Ghd7* delays heading regardless of *Hd1* status—and more strongly when *Hd1* is functional [30]. Here, we find parallel regulatory patterns for the heading date and photosynthesis: the FN genotype with the shortest heading date had the highest Pn (21.36 μmol CO_2_ m^−2^ s^−1^), while the FF genotype with the longest heading date had the lowest Pn (12.62 μmol CO_2_ m^−2^ s^−1^) (Figure 2A). Similarly, NN headed earlier and exhibited a higher Pn than NF; the same trend held for the Gs, Ci, and Tr (Figure 2B–D). Thus, the following holds under the LD condition: (i) functional *Hd1* enhances photosynthesis only when *ghd7* is non-functional; (ii) functional *Ghd7* suppresses photosynthesis regardless of *Hd1* status—and more strongly when *Hd1* is functional. These results demonstrate that heading date genes may directly regulate photosynthetic capacity.

In the Z43 and Z44 populations, *Hd1* exhibited significant genetic effects on the chlorophyll contents (Table 1 and Table 2). However, no significant effect of *Hd1* on the photosynthesis-related traits was observed in the Z44 population. Specifically, in the Z43 population, the *R*^2^ values for the chlorophyll *a*/*b* contents explained by *Hd1* were 17.60% and 27.62%, respectively, which were significantly higher than the corresponding values in the Z44 population (9.81% and 13.98%, respectively). Although the functional *Hd1* allele positively regulated the chlorophyll contents in both populations, its genetic effect intensity was obviously weakened in the Z44 population (Table 1 and Table 2). Background genotype analysis revealed that all NILs in the Z43 population carried functional *Ghd7* alleles, and their overall genetic background was highly consistent with the NILs of *Hd1*^ZS97^*Ghd7*^MY46^ and *hd1*^MY46^*Ghd7*^MY46^ in the Z44 population. Further allele combination analysis in the Z44 population showed that the functional *Hd1* allele significantly reduced the Pn (Figure 2A) under a functional *Ghd7* background, consistent with the trend observed in the Z43 population. In summary, *Hd1*’s genetic regulation of the photosynthetic parameters and chlorophyll content is contingent upon *Ghd7* functionality. Transcriptome comparative analysis further demonstrated that functional *Ghd7* significantly downregulates the expression of multiple key genes in the chlorophyll biosynthesis pathway, and this repression is potentiated in the presence of a functional *Hd1* allele. Therefore, *Hd1*’s regulatory influence on these physiological traits may depend on functional *Ghd7*.

This study reveals that *Hd1* and *Ghd7* jointly regulate photosynthesis through epistatic interaction. Functional *Hd1* increases the chlorophyll content but reduces the photosynthetic rate under LD conditions (Table 2), while functional *Ghd7* suppresses both chlorophyll accumulation and key photosynthetic parameters (Pn, Gs, Ci, Tr) (Table 1). Their interaction is critical: *Ghd7*’s inhibitory effect on photosynthesis is strongly enhanced by functional *Hd1*, and *Hd1* only boosts photosynthesis in a *ghd7* loss-of-function background (Figure 2). Transcriptome analysis confirms that *Ghd7* represses photosynthesis- and chloroplast-related genes, primarily in the presence of functional *Hd1*. Although *Hd1* enhances the chlorophyll content, it concurrently diminishes the actual photosynthetic efficiency, indicating that chlorophyll accumulation and photosynthetic performance are under distinct regulatory control—likely mediated by divergent molecular mechanisms.

## 4. Materials and Methods

### 4.1. Plant Material

Two NIL populations were used in this study. One of them was segregated at both the *Hd1* and *Ghd7* loci. The development process is illustrated in Figure 1 and described below. One F_9_ plant from the ZS97/MY46 cross was backcrossed with MY46 for two generations. One BC_2_F_1_ plant heterozygous at both the *Hd1* and *Ghd7* loci was identified and selfed. In the resulting BC_2_F_2_ population, a plant heterozygous for both genes was selected and selfed. The resultant BC_2_F_3_ population was genotyped using functional or closely linked DNA markers for the two loci: the STS marker Si9337 for detecting the *Hd1* locus and the SSR marker RM5436 for detecting the *Ghd7* locus. A total of 50 plants homozygous at the *Hd1* and/or *Ghd7* loci were identified and selfed. One NIL population, namely Z44, comprising 10, 7, 12, and 21 lines of *hd1*^MY46^*ghd7*^ZS97^, *Hd1*^ZS97^*ghd7*^ZS97^, *hd1*^MY46^*Ghd7*^MY46^, and *Hd1*^ZS97^*Ghd7*^MY46^, respectively, was developed.

The other NIL population only segregated at the *Hd1* locus. One BC_2_F_3_ single plant segregating only at the *Hd1* locus was selected. This plant was selfed to a BC_2_F_4_ population, which was assayed with a functional DNA marker of *Hd1*. A total of 48 BC_2_F_4_ plants homozygous at the *Hd1* locus were identified and selfed. From these, one NIL population Z43 was developed, comprising 26 lines of *hd1*^MY46^ and 22 lines of *Hd1*^ZS97^, respectively.

### 4.2. Field Experiments and Phenotyping

The rice populations were tested at the China National Rice Research Institute located in Hangzhou, Zhejiang province, China. During the period of floral transition in the rice materials tested, the day length in Hangzhou corresponded to LD conditions. The planting density was 16.7 cm × 26.7 cm in all the trials. Field management followed the normal agricultural practice. The experiments followed a randomized complete block design with two replications, and in each replication, one line was grown in a single row of ten plants.

For chlorophylls *a* and *b*, flag leaves at the heading stage were sampled and stored at −80 °C, and the detached leaves were soaked in a mixed liquid of ether, acetone, and water (V:V:V = 4.5:4.5:1) in the dark for 24 h and then measured at 645 and 663 nm using a Beckman spectrophotometer. Finally, the chlorophyll *a* and *b* contents were calculated [35]. For the Pn, Gs, Ci, and Tr, the flag leaf parameters were measured using a portable photosynthesis system (LI-6400, Li-Cor Inc., Lincoln, NE, USA). Flag leaves at the heading stage were detected on clear days between 9:00 and 11:00 a.m., when photosynthetically active radiation (PAR) at the leaf surface was controlled at 1500 μmol photons m^−2^ s^−1^, the CO_2_ concentration at 400 µM/mol, the flow rate at 500 µM/s, and the temperature at 30 °C. Mean values across three replications were taken for the data analysis.

### 4.3. RNA-Seq Analysis

Flag leaves at the heading stage were sampled at 9:00 a.m. and stored at −80 °C with three biological replicates per group. Total RNA was extracted using the TRIzol reagent (Invitrogen, Carlsbad, CA, USA), according to the manufacturer’s protocol. RNA quantification and qualification, library preparation for transcriptome sequencing, clustering and sequencing, data analysis, and quality control were conducted by LianChuan (http://www.lc-bio.com/), according to their protocol. Differential expression analysis between the two genotypic groups was performed using the DESeq R package (version 1.10.1) with a model based on the negative binomial distribution. The resulting *p* values were adjusted for controlling the false discovery rate, using the Benjamini–Hochberg approach. Genes with an adjusted *p*-value < 0.01 and log2|fold change| ≧ 1.0 were designated as DEGs.

### 4.4. Data Analysis

Two-way ANOVA was conducted to test the main and epistatic effects, and Duncan’s multiple range test was used to examine the phenotypic differences between genotypic groups. The analysis was performed using the SAS version 8.2 procedure GLM [36].

## Figures and Tables

**Table 1:** The *Hd1* and *Ghd7* effects on the six photosynthetic physiological traits detected in the Z44 population.

Traits ^a^	*Hd1*			*Ghd7*			*Hd1*×*Ghd7*		
	*p* Value ^b^	*A* ^c^	*R*^2^ % ^d^	*p* Value	*A*	*R*^2^ %	*p* Value	I-Effect ^e^	*R*^2^ %
Chl*a*	0.0010	−0.07	9.81	<0.0001	−0.13	14.32	0.0525		
Chl*b*	0.0001	−0.04	13.98	0.0097	−0.02	6.22	0.2684		
Pn	0.1128			<0.0001	−3.31	35.14	0.0016	−5.03	5.58
Gs	0.0706			<0.0001	−0.19	43.15	0.0001	−0.36	7.84
Ci	0.4607			<0.0001	−42.99	53.13	<0.0001	−60.46	6.69
Tr	0.0650			<0.0001	−1.78	40.37	<0.0001	−4.06	10.7

^a^ Chl*a*, chlorophyll *a* (mg/g·FW); Chl*b*, chlorophyll *b* (mg/g·FW); Pn, photosynthesis rate (μmol CO_2_ m^−2^ s^−1^); Gs, stomatal conductance (mol H_2_O m^−2^ s^−1^); Ci, intercellular CO_2_ concentration (μmol CO_2_ m^−2^ s^−1^); Tr, transpiration rate (mmol H_2_O m^−2^ s^−1^); ^b^ Two-way ANOVA; ^c^ *A*, additive effect of replacing Milyang 46 allele with Zhenshan 97 allele. ^d^ *R*^2^ (%), proportion of phenotypic variance explained by QTL effect. *R*^2^ = (V_G_/V_P_) × 100%, V_G_, genotypic variance; V_P_, Total phenotypic variance; ^e^ I-effect, positive value: parental type < recombinant type; negative value: parental type > recombinant type.

**Table 2:** The effects of *Hd1* on the six photosynthetic physiological traits detected in the Z43 population.

Traits ^a^	Phenotype (Mean ± SD)		*p* Value	*A* ^b^	*R*^2^ % ^c^
	ZS97	MY46			
Chl*a*	3.71 ± 0.47	3.42 ± 0.28	<0.0001	−0.15	17.6
Chl*b*	1.08 ± 0.12	0.98 ± 0.08	<0.0001	−0.05	27.62
*P*_n_	15.03 ± 3.83	20.65 ± 2.40	<0.0001	2.81	31.9
*G*_s_	0.19 ± 0.11	0.35 ± 0.21	0.0011	0.08	10.77
*C*_i_	235.45 ± 31.03	280.43 ± 26.61	<0.0001	22.49	21.19
*T*_r_	2.86 ± 1.06	4.25 ± 1.83	0.0009	0.69	11.22

^a^ Chl*a*, chlorophyll *a* (mg/g·FW); Chl*b*, chlorophyll *b* (mg/g·FW); Pn, photosynthesis rate (μmol CO_2_ m^−2^ s^−1^); Gs, stomatal conductance (mol H_2_O m^−2^ s^−1^); Ci, intercellular CO_2_ concentration (μmol CO_2_ m^−2^ s^−1^); Tr, transpiration rate (mmol H_2_O m^−2^ s^−1^); ^b^ *A*, additive effect of replacing Milyang 46 allele with Zhenshan 97 allele. ^c^ *R*^2^ (%), proportion of phenotypic variance explained by QTL effect. *R*^2^ = (V_G_/V_P_) × 100%, V_G_, genotypic variance; V_P_, total phenotypic variance.

**Table 3:** The results of the transcriptome analysis among the different comparison groups.

Population	Group ^a^	Total DEGs	Upregulated Genes	Downregulated Genes
Z43	FF vs. NF	1805	523	1282
Z44	FF vs. FN	2836	920	1916
	NF vs. NN	1097	476	621
	FN vs. NN	564	302	262
	FF vs. NF	2082	737	1345

^a^ FF, *Hd1*^ZS97^*Ghd7*^MY46^; FN, *Hd1*^ZS97^*ghd7*^ZS97^; NF, *hd1*^MY46^*Ghd7*^MY46^; NN, *hd1*^MY46^*ghd7*^ZS97^; “F” = functional allele; “N” = non-functional allele.

## Data Availability

The original contributions presented in the study are included in the article/Supplementary Materials; further inquiries can be directed to the corresponding authors.

## Supplementary Materials

The following supporting information can be downloaded at https://www.mdpi.com/article/10.3390/plants15060977/s1. Table S1: DEGs associated with photosynthesis and chloroplast development in the significantly enriched GO terms from the *Hd1*^ZS97^ vs. *hd1*^MY46^ group in Z43. Table S2: DEGs associated with photosynthesis and chloroplast development in the significantly enriched GO terms from the four *Hd1*-*Ghd7* genotype combinations in Z44.
