# Supplementary material for: Genetic Interaction Effects of Heading Date Genes Hd1 and Ghd7 on Photosynthetic Traits at the Heading Stage in Rice
Source: Plants (Basel). 2026 Mar 22;15(6):977. doi: 10.3390/plants15060977 (PMC13030060; doi:10.3390/plants15060977)
Supplement: Supplementary file 1 [file plants-15-00977-s001.zip › plants-4194783-supplementary.pdf]

Table S1. DEGs associated with photosynthesis and chloroplast development in the significantly enriched GO terms from the *Hd1*^{ZS97} vs *hd1*^{MY46} group in Z43

Gene ID	log2(fc)	Gene name	Description
LOC_Os04g51350	-inf	*MPR25*	pentatricopeptide
LOC_Os07g02280	inf		pentatricopeptide
LOC_Os08g04450	-1.30		DAG protein, chloroplast precursor
LOC_Os07g08160	-1.95		early light-induced protein, chloroplast precursor
LOC_Os09g36200	-1.58	*SGR*	senescence-inducible chloroplast stay-gree n protein 1,
LOC_Os02g37060	-4.22		photosystem II 5 kDa protein, chloroplast precursor,
LOC_Os02g36850	-2.00		oxygen evolving enhancer protein 3, identical,
LOC_Os07g01480	-1.70		oxygen evolving enhancer protein 3 domain containing protein

Note: Only the differentially expressed genes in the significantly enriched GO terms associated with photosynthesis and chloroplast development were shown.

Table S2 DEGs associated with photosynthesis and chloroplast development in the significantly enriched GO terms from the four *Hd1-Ghd7* genotype combinations in Z44.

Group	Gene ID	log2(fc)	Gene name	Description
FF vs FN	LOC_Os04g51350	inf	*MPR25*	pentatricopeptide
	LOC_Os03g22780	inf	*OsDVR*	DVR
	LOC_Os03g03990	-inf	*OscpSRP43*	signal recognition particle 43 kDa protein, chloroplast precursor
	LOC_Os11g16550	-inf	*OsGUN4*	uncharacterized protein ycf53
	LOC_Os04g58200	-4.24	*OsPORA*	protochlorophyllide reductase A, chloroplast precursor
	LOC_Os03g45400	-3.44	*OsNUS1*	antitermination NusB domain-containing protein
	LOC_Os03g27770	-3.36	*OsHO2*	heme oxygenase 2
	LOC_Os01g01340	-3.35	*OsLIR1*	light-induced protein 1-like
	LOC_Os04g51280	-3.25	*WSP1*	DAGprotein,chloroplastprecursor;expressed protein
	LOC_Os02g02860	-2.92	*OsGluRS*	glutamyl-tRNA synthetase
	LOC_Os05g49920	-2.80	*OsPPR6*	pentatricopeptide
	LOC_Os07g47300	-2.28	*Obgc*	spo0B-associated GTP-binding protein
	LOC_Os03g19510	-2.25	*OsClpP5*	OsClp4 - Putative Clp protease homologue
	LOC_Os01g48380	-2.20	*ALS3*	PPR repeat domain containing protein
	LOC_Os10g35370	-2.06	*OsPORB*	oxidoreductase, short chaindehydrogenase/reductase family domain containing family
	LOC_Os05g34040	-1.93	*TCD5*	monooxygenase
	LOC_Os03g31150	-1.75	*AL1*	expressed protein
	LOC_Os01g17170	-1.1	*YGL8*	magnesium-protoporphyrin IX monomerthyl ester cyclase, chloroplast precursor
	LOC_Os01g01280	1.38	*OsTLP27*	expressed protein
	LOC_Os03g03370	1.53	*DSM2*	fatty acid hydroxylase
	LOC_Os09g36200	2.7	*SGR*	senescence-inducible chloroplast stay-green protein 1
	LOC_Os04g59440	3.28	*psbS2*	chlorophyll A-B binding protein
**NF vs NN**	LOC_Os05g49920	-2.31	*OsPPR6*	pentatricopeptide
	LOC_Os03g45400	-1.41	*OsNUS1*	antitermination NusB domain-containing protein
	LOC_Os06g26234	-1.28	*OsBE1*	1,4-alpha-glucan-branching enzyme
	LOC_Os03g27770	-1.12	*OsHO2*	heme oxygenase 2
	LOC_Os01g01340	1.4	*OsLIR1*	light-induced protein 1-like
	LOC_Os03g15840	1.71	*pls2*	glycosyl transferase, group 1 domain containing protein
**FN vs NN**	LOC_Os11g16550	inf	*OsGUN4*	uncharacterized protein ycf53
	LOC_Os09g30330	-2.62		photosystem I reaction center subunit,chloroplast precursor; phosphatidylinositol transfer
	LOC_Os07g08150	-1.69		early light-induced protein, chloroplast precursor
	LOC_Os07g09190	1.19		transketolase
	LOC_Os10g35840	2.38		shikimate/quinate 5-dehydrogenase
	LOC_Os07g32890	3.86		ATP synthase gamma chain, putative, expressed; 3'-5' exonuclease,
**FF vs NF**	LOC_Os04g58200	-4.07	OsPORA	protochlorophyllide reductase A,

			chloroplast precursor
LOC_Os01g01340	-3.99	*OsLIR1*	light-induced protein 1-like
LOC_Os03g27770	-2.65	*OsHO2*	heme oxygenase 2
LOC_Os03g45400	-2.58	*OsNUS1*	antitermination NusB domain-containing protein
LOC_Os02g02860	-2.55	*OsGluRS*	glutamyl-tRNA synthetase
LOC_Os04g51280	-2.39	*WSP1*	DAG protein, chloroplast precursor; expressed protein
LOC_Os07g47300	-2.28	*Obgc*	spo0B-associated GTP-binding protein
LOC_Os03g19510	-2.07	*OsClpP5*	OsClp4 - Putative Clp protease homologue
LOC_Os01g48380	-1.97	*ALS3*	PPR repeat domain containing protein
LOC_Os10g35370	-1.56	*OsPORB*	oxidoreductase, short chain dehydrogenase/reductase family domain containing family
LOC_Os03g31150	-1.4	*AL1*	expressed protein
LOC_Os09g36200	1.09	*SGR*	senescence-inducible chloroplast stay-green protein 1
LOC_Os01g64960	1.21	*psbS2*	chlorophyll A-B binding protein
LOC_Os01g01280	1.92	*OsTLP27*	expressed protein

Note: Only the differentially expressed genes in the significantly enriched GO terms associated with photosynthesis and chloroplast development were shown. FF, $Hd1^{ZS97}Ghd7^{MY46}$; FN, $Hd1^{ZS97}ghd7^{ZS97}$; NF, $hd1^{MY46}Ghd7^{MY46}$; NN, $hd1^{MY46}ghd7^{ZS97}$.
